# Oscillation of Autophagy Induction under Cellular Stress and What Lies behind It, a Systems Biology Study

**DOI:** 10.3390/ijms24087671

**Published:** 2023-04-21

**Authors:** Bence Hajdú, Luca Csabai, Margita Márton, Marianna Holczer, Tamás Korcsmáros, Orsolya Kapuy

**Affiliations:** 1 Department of Molecular Biology, Institute of Biochemistry and Molecular Biology, Semmelweis University, 1085 Budapest, Hungary; 2 Earlham Institute, Norwich Research Park, Norwich NR4 7UG, UK; 3 Department of Genetics, Eötvös Loránd University, 1117 Budapest, Hungary; 4 Quadram Institute Bioscience, Norwich Research Park, Norwich NR4 7UQ, UK; 5 Department of Metabolism, Digestion and Reproduction, Imperial College London, London W12 0NN, UK

**Keywords:** feedback loops, autophagy, bistable switch, oscillation, mathematical modelling

## Abstract

One of the main inducers of autophagy-dependent self-cannibalism, called ULK1, is tightly regulated by the two sensor molecules of nutrient conditions and energy status, known as mTOR and AMPK kinases, respectively. Recently, we developed a freely available mathematical model to explore the oscillatory characteristic of the AMPK-mTOR-ULK1 regulatory triangle. Here, we introduce a systems biology analysis to explain in detail the dynamical features of the essential negative and double-negative feedback loops and also the periodic repeat of autophagy induction upon cellular stress. We propose an additional regulatory molecule in the autophagy control network that delays some of AMPK’s effect on the system, making the model output more consistent with experimental results. Furthermore, a network analysis on AutophagyNet was carried out to identify which proteins could be the proposed regulatory components in the system. These regulatory proteins should satisfy the following rules: (1) they are induced by AMPK; (2) they promote ULK1; (3) they down-regulate mTOR upon cellular stress. We have found 16 such regulatory components that have been experimentally proven to satisfy at least two of the given rules. Identifying such critical regulators of autophagy induction could support anti-cancer- and ageing-related therapeutic efforts.

## 1. Introduction

Autophagy-dependent self-cannibalism is an evolutionarily conserved dynamic catabolic process in eukaryotic cells where double-membrane vesicles containing cytoplasmic components are formed and degraded in special vesicles [1,2]. Autophagy plays an important role in maintaining cellular homeostasis [1] and contributes to cell survival by breaking down and recycling cytoplasmic components [3]. It can provide precursors and energy to the cell during starvation and other stress responses, such as endoplasmic reticulum stress [4,5].

The key controller of autophagy initiation is ULK1 (unc-51 like autophagy activating kinase 1), which is part of the family of kinases ULK1-4 in humans. ULK1 activity is directly controlled by the proper balance between mTOR (mammalian target of rapamycin) and AMPK (AMP-activated kinase). mTOR is a serine/threonine kinase involved in the regulation of cellular proteostasis as part of the mTORC1 (mammalian target of rapamycin complex 1) [6,7]. AMPK is a kinase that detects the depletion of ATP and the increase in AMP and promotes degradation processes [8,9]. These two proteins oppositely regulate ULK1 [10,11]. Under nutrient supply, mTORC1 inhibits the ULK complex via phosphorylation of ULK1 [10,11]. mTORC1 can down-regulate AMPK, which excludes the activation of autophagy [12,13]. However, AMPK is activated under energy-poor conditions and induces self-digestion by phosphorylating ULK1 and inhibiting mTORC1 [10,11]. The autophagic response is ”fine-tuned“ by two different ULK1-dependent inhibiting mechanisms. On the one hand, ULK1 can negatively regulate AMPK by phosphorylation and thus reduce its activity [14]. On the other hand, ULK1 phosphorylates the RAPTOR subunit of mTORC1, resulting in the inhibition of mTORC1 in the presence of persistent nutrient deficiency [15,16].

ULK1 alone is not sufficient to induce autophagy but forms a so-called ULK1 complex containing ULK1, FIP200 and ATG13 proteins. The ULK complex then activates the VPS34-Beclin1-ATG9 complex (also known as PI3K complex), which promotes phagophore formation [1,2,17,18]. With the growth of the phagophore, substrates enter into the double-membrane vesicle, which is called the autophagosome. Once an autophagosome is formed, it must fuse with the lysosome to become an autophagolysosome and its contents can be degraded [17,19]. The main steps of autophagy are managed by difficult protein complexes and ubiquitin-like conjugation systems as well [2]. These will not be discussed in detail here as our study focuses on the initiation step of autophagy.

Autophagy plays an important role in cell metabolism, ageing and longevity. Autophagy activation may contribute to lifespan extension, while autophagy function decreases with age [3,20,21]. Abnormalities in autophagy function have been observed in the pathogenesis of many human diseases [22], such as neurodegenerative and metabolic diseases [5,23], cancer [23,24], various infections [5,25] and inflammatory bowel disease [26,27,28]. In the pathology of autophagy-related diseases, there are two possibilities: one is if the process is not properly induced (e.g., in neurodegenerative diseases) and the other if it is not properly inactivated (e.g., in tumour progression). In addition, both suppression and enhancement of autophagy can promote the development and growth of cancer through different pathways [29]; thus, the possible therapeutic strategies can be the use of autophagy-activating agents and/or autophagy-inhibiting agents during treatment [22,29]. Therefore, in the case of many diseases, exact knowledge on the initiation of autophagy can be crucial in finding therapeutic targets.

The induction of autophagy can be influenced by drugs that modify the balance between AMPK and mTORC1, thus activating or inhibiting the autophagy initiator ULK1 [11]. One commonly used compound is rapamycin, which targets the mTOR subunit of the mTORC1 as an allosteric inhibitor in a complex with FKBP12 (12-kDa FK506-binding protein) [7,30]. Thus, rapamycin promotes the activation of AMPK and ULK1, and the induction of autophagy [11]. Several rapamycin analogues (rapalogs) have been developed because of their improved solubility and pharmacokinetics [30].

Autophagy can also be induced by anti-cancer agent HDAC (histone deacetylase)-inhibiting compounds [31]. SAHA (suberoylanilide hydroxamic acid, vorinostat), the first HDAC inhibitor approved for cutaneous T-cell lymphoma treatment, increases LC3 expression and inhibits mTORC1 leading to ULK1 activation and autophagy induction [32].

In this study, we introduce a theoretical analysis to explore the dynamical characteristic of both negative and double-negative feedback loops of ULK1-controlled autophagy induction. In our analysis, we pay particular attention to the understanding of how the periodic activation of autophagy operates with the help of AMPK and mTOR kinases upon cellular stress. We suggest an additional control molecule (called “regulator”) in the autophagy control network acting like the key regulator of autophagy induction. By using bioinformatics resources, we identify more than 1000 proteins with similar direct or indirect connections that could later become important therapeutic targets in various diseases.

## 2. Results

### 2.1. Time-Delayed Negative Feedback Loop Results in Periodic Activation of Autophagy upon mTOR Inhibition

Our goal here is to explain the dynamical features of both negative and double-negative feedback loops of ULK1-controlled autophagy induction. By using systems biology methods, we recently confirmed that autophagy induction has an oscillatory characteristic upon various cellular stress events [33,34,35]. We proved both experimentally and theoretically that nutrient deprivation or mTOR (here, mTOR refers to mTORC1) inhibition via rapamycin treatment resulted in the periodic repeat of ULK1 activation and inactivation, and created an oscillatory characteristic of autophagy [33]. This could be explained by the fact that to use the building blocks created by autophagy, mTOR needs to be turned back on to restart the anabolic processes again and again.

**Figure 1:**
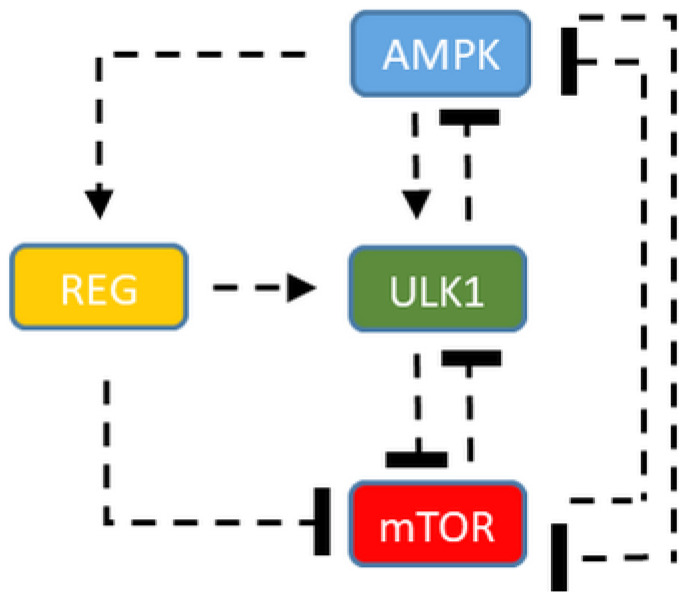
The characteristic of the time-delayed ULK1-mTORC1-AMPK regulatory triangle. The simple wiring diagram of autophagy induction controlled by an extra regulatory protein (REG). Dashed lines show how the molecules can influence each other. Blocked end lines denote inhibition.

In our model, AMPK—as a so called ”pre-activator“—is able to promote autophagy initiation by phosphorylating ULK1, while ULK1 down-regulates AMPK via phosphorylation [10,14,36,37,38]. Therefore, the core of the control network is the negative feedback loop between AMPK and ULK1 (Figure 1). Besides this negative feedback loop, two double-negative feedback loops are also present, with one between ULK1 and mTOR, while AMPK and mTOR are also mutually exclusive. To generate a so-called ”delay“ in the negative feedback loop between AMPK and ULK1, here, we built up an additional protein (called “regulator”—REG) in the regulatory network that gets activated by AMPK (Figure 1). Besides, ULK1 becomes up-regulated by REG, generating an AMPK-dependent indirect activation of autophagy via REG. Since the AMPK -> ULK1 direct connection is also proved [10,37,38], it is also present in the control network; however, we assume that the positive connection via REG is stronger (Figure 1). To further analyse the positive role of REG in autophagy induction, here, we suggest that the regulator is also able to inhibit mTOR.

**Figure 2:**
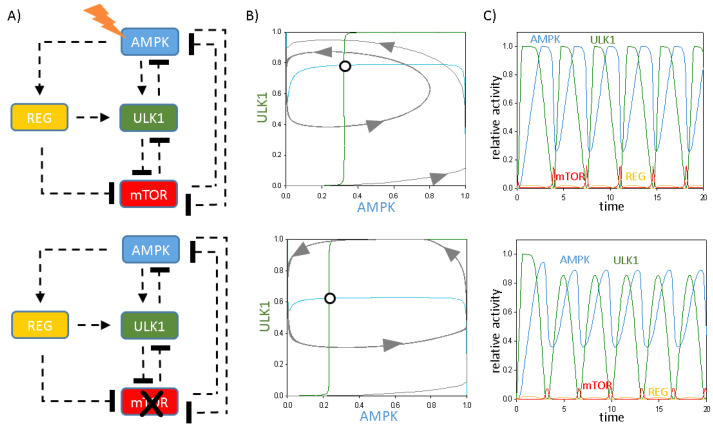
The characteristic of the time-delayed ULK1-mTOR-AMPK regulatory triangle upon cellular stress. (**A**) The simple wiring diagram of autophagy induction controlled by an extra protein upon (**upper panel**) cellular stress (stress = 0.5) or (**lower panel**) rapamycin treatment (mTORT = 0.5). Dashed lines show how the molecules can influence each other. Blocked end lines denote inhibition. (**B**) Phase plane diagrams are plotted upon (**upper panel**) cellular stress (stress = 0.5) or (**lower panel**) rapamycin treatment (mTORT = 0.5). The balance curves of ULK1 (green) and AMPK (blue) are plotted. Intersections of nullclines represent unstable (unfilled circle) steady states. Trajectories are depicted with dotted grey lines. (**C**) The temporal dynamics is simulated under (**upper panel**) cellular stress (stress = 0.5) or (**lower panel**) rapamycin treatment (mTORT = 0.5). The relative activity of mTOR, AMPK, ULK1 and REG is shown.

The abovementioned four conditions required for oscillation are fulfilled not only under cellular stress but also when mTOR is inhibited by rapamycin (Figure 2A). To illustrate the dynamical characteristic of the negative feedback loop between AMPK and ULK1, we plotted phase plane diagrams, where ordinary differential equations are written for dAMPK/dt and dULK1/dt both in cellular stress and rapamycin treatment (Figure 2B). Then, a coordinate system is spanned by ULK1 and AMPK, and the so-called nullclines—namely, dULK1/dt = 0 (green) and dAMPK/dt = 0 (blue)—are depicted. Where the nullclines intersect each other, the system might be in a steady state. Under physiological conditions, the stress signal is zero. In this case, there is only one stable state, called ”Phys.cond.“ with high levels of AMPK and ULK1 (data not shown). As the stress increases in the cell, an unstable steady state is formed surrounded with a stable limit cycle (see unfilled circle and grey trajectories in Figure 2B). Computer simulations also confirm that although mTOR is knocked out of the network (directly or indirectly via AMPK activation), oscillations of autophagy initiation are observed through the delayed negative feedback loop of AMPK -> “regulator” -> ULK1 –| AMPK (Figure 2C), which is consistent with our previous experimental results [33,34].

We claim that, in this way, the system might have an opportunity to release energy and utilise the building blocks produced from more complex biological elements via autophagy. It seems to be essential to remove the damaged elements and re-utilise the unnecessary elements; otherwise, the cell has to commit early cell death.

### 2.2. Biological Importance of Bistable Switch at Autophagy Induction

**Figure 3:**
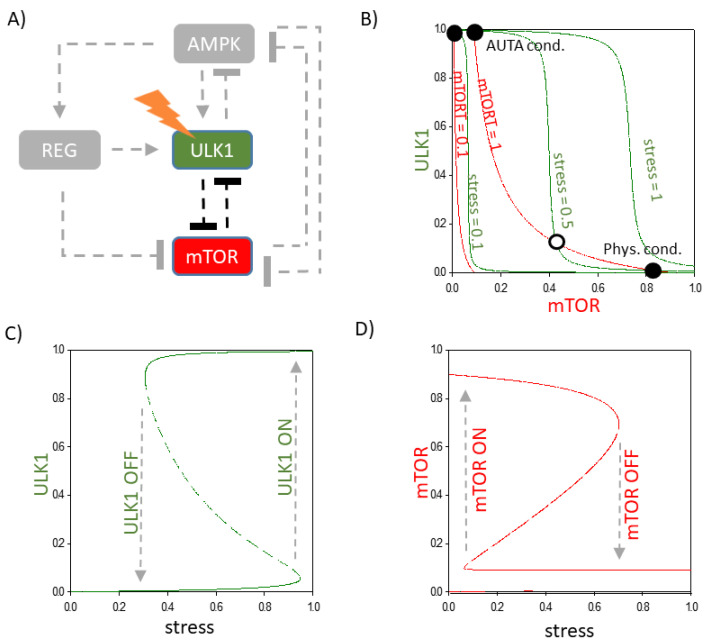
The characteristic of double-negative feedback between ULK1 and mTOR. (**A**) Wiring diagram of the ULK1-mTOR double-negative feedback loop. Dashed lines show how the molecules can influence each other. Blocked end lines denote inhibition. Grey colour means that the connection and component are removed from the network. (**B**) Phase plane diagrams are plotted upon various levels of stress. The balance curves of ULK1 (green) and mTOR (red) are plotted. The phase plane is shown for stress = 0.1, 0.5, 1 and mTORT = 0.1, 1. Intersections of nullclines represent the stable (filled circle) and unstable (unfilled circle) steady states. Signal response curves of (**C**) ULK1 and (**D**) mTOR are shown. The solid lines in the signal response curve denote stable states, while dashed lines depict the unstable state.

Although the delayed negative feedback loop is essential for oscillation, the question immediately arises as to the role of double-negative feedback loops (i.e., ULK1 -| mTOR -|ULK1, AMPK -|mTORC1 -|AMPK) in autophagy induction. To find this out, the connection between the activator of autophagy (ULK1) and the inhibitor of autophagy (mTOR) is first examined in more detail. For this, other components of the control network and regulatory loops are eliminated (see grey lines and components in Figure 3A).

To illustrate the dynamical characteristic of the double-negative feedback loop between mTOR and ULK1, we plotted phase plane diagrams, where the nullclines (green line: dULK1/dt = 0; red line: dmTOR/dt = 0) are depicted (Figure 3B). Under physiological conditions, there is only one stable state called ”Phys. cond.“ with high levels of mTOR and low levels of ULK1. As the stress increases in the cell, two stable steady states are formed, called ”Phys. cond.“ (high mTOR and low ULK1) and ”AUTA cond.“ (high ULK1 and low mTOR), separated by an unstable one (see unfilled circle in Figure 3B). Since at physiological condition the system chooses the ”Phys. cond.“ steady state, the system is not able to turn on autophagy under a critically low level of cellular stress. However, as the stress increases, the lower steady state disappears and the control system is forced to transition to the upper steady state where ULK1 becomes active (see the black dot of ”AUTA cond.“ in Figure 3B). In case of rapamycin-dependent down-regulation of mTOR, the nullcline of mTOR moves left (see the red curve, when mTORT = 0.1 in Figure 3B); therefore, a complete diminishing of the ”Phys.cond.“ steady state can be observed and the cell quickly enters autophagy initiation with active ULK1 even at S = 0.

This double-negative feedback between mTOR and ULK1 could potentially provide a threshold for autophagy activation. The signal response curves demonstrate that the cellular stress level has to reach a critical threshold to turn on ULK1-dependent autophagy (see the grey dashed arrows in Figure 3C,D). Before the transition, the stress level is low; therefore, the inhibitor (mTOR) overwhelms the activator (ULK1)—namely, the mTOR level is still high enough to block the activation of autophagy. However, as soon as the stress level reaches a critical value, the switch-like induction of ULK1 activator helps to eliminate the mTOR inhibitor, resulting in a toggle switch that guarantees the proper activation of autophagy initiation (see the grey dashed arrows in Figure 3C,D).

We assume that autophagy induction (i.e., mTOR inactivation and ULK1 activation) is reversible, namely, if the stress level drops back to zero (i.e., the conditions become similar to the physiological ones), the regulatory system is not locked in the autophagic state, rather it goes back to its original state when mTOR was active and ULK1 was inactive (see the grey dotted arrows in Figure 3C,D). We claim that this reversible activation of autophagy initiation guarantees that the molecules of autophagy are active only when the stress level reaches a critical value. It also makes possible mTOR-induced protein synthesis by using those components that were digested by autophagy previously.

### 2.3. A Direct Negative Feedback Loop of ULK1 Cannot Explain the Periodic Repeat of Autophagy Induction upon Cellular Stress

**Figure 4:**
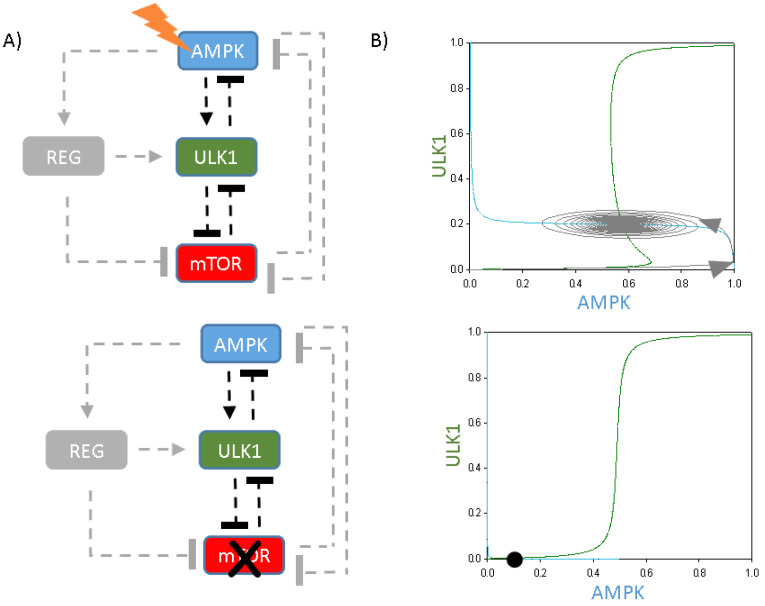
The introduction of the negative feedback loop between ULK1 and AMPK. (**A**) The wiring diagram of the ULK1-AMPK-mTOR regulatory network upon (**upper panel**) cellular stress (stress = 5) or (**lower panel**) rapamycin treatment (mTORT = 0.01). Dashed lines show how the molecules can influence each other. Blocked end lines denote inhibition. Grey colour means the connection and component are removed from the network. (**B**) Phase plane diagrams are plotted upon (**upper panel**) starvation (stress = 5) or (**lower panel**) rapamycin treatment (mTORT = 0.01). The balance curves of ULK1 (green) and AMPK (blue) are plotted. Intersections of nullclines represent the stable (filled circle) and unstable (unfilled circle) steady states. Trajectories are depicted with dotted grey lines.

To further analyse the importance of the ULK1-mTOR double-negative feedback loop upon periodic repetition of self-cannibalism, this core connection was extended with a simple, direct ULK1-AMPK negative feedback loop (see the coloured components and connections in Figure 4A). Although the ULK1-AMPK negative feedback loop was observed, no stable limit cycle oscillation was detected on the phase plane diagram spanned by ULK1 (green) and AMPK (blue) (Figure 4B). Under physiological conditions, the nullclines intersect close to zero, suggesting that both AMPK and ULK1 are down-regulated (data not shown). Although the increasing level of cellular stress caused the AMPK curve to move upwards and intersect the S-shaped ULK1 balance curve at an unstable point, only a damped oscillation of autophagy induction was observed (Figure 4B, upper panel). Thus, a double-negative feedback combined with a direct negative feedback loop is not stable enough for a limit cycle oscillation upon cellular stress.

Logically, in the absence of mTOR (by simulating rapamycin treatment), the cell could not promote autophagy induction at all (Figure 4B, lower panel). Since mTOR inhibition completely diminishes the double-negative feedback loop between ULK1 and mTOR, rapamycin treatment causes a homeostatic response due to the direct negative feedback loop between AMPK and ULK1, with low levels of AMPK and ULK1 (Figure 4B, lower panel).

Corresponding with our previous data, these analyses further confirm that a direct negative feedback loop combined with a double-negative feedback loop is not able to guarantee the oscillatory characteristic of autophagy induction upon various cellular stress events.

### 2.4. The Direct AMPK-ULK1-mTOR Regulatory Triangle Cannot Explain the Periodic Repeat of Autophagy Induction during Rapamycin Treatment

**Figure 5:**
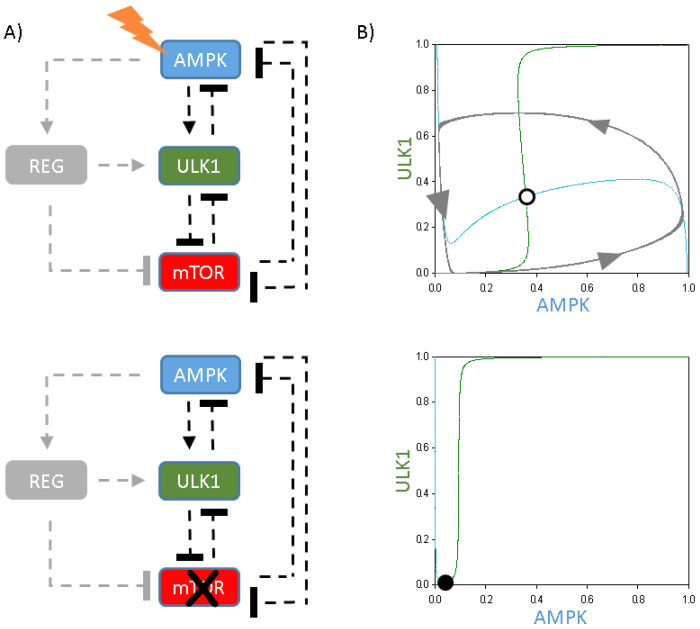
The characteristic of the ULK1-mTOR-AMPK regulatory triangle. (**A**) The wiring diagram of ULK1-AMPK-mTOR regulatory network upon (upper panel) cellular stress (stress = 3) or (lower panel) rapamycin treatment (mTORT = 0.01). Dashed lines show how the molecules can influence each other. Blocked end lines denote inhibition. Grey colour means that the connection and component are removed from the network. (**B**) Phase plane diagrams are plotted upon (upper panel) cellular stress (stress = 3) or (lower panel) rapamycin treatment (mTORT = 0.01). The balance curves of ULK1 (green) and mTORC1 (red) are plotted. Intersections of nullclines represent the stable (filled circle) and unstable (unfilled circle) steady states. Trajectories are depicted with dotted grey lines.

It is well known that AMPK is able to down-regulate mTOR activity, but it has been recently shown that mTOR also inhibits AMPK generating another double-negative feedback loop in the control network [12,13]. Now, the heart of the network contains a regulatory triangle where a direct negative feedback loop gets stabilised by two double-negative feedback loops (see the coloured lines and components in Figure 5A). Upon cellular stress, the autophagy ”pre-activator“ (AMPK) helps to activate the main activator of autophagy initiation (ULK1) by eliminating its antagonist (mTOR) and the cellular transition occurs (i.e., switch from physiological to stress state) (Figure 5B). Subsequently, the pre-activator (AMPK) gets down-regulated by the activator (ULK1); meanwhile, between the pre-activator (AMPK) and the inhibitor (mTOR), a bistable switch is generated. This so-called amplified negative feedback loop causes a significant qualitative change on the phase plane upon cellular stress (Figure 5B, upper panel). In this case, the S- and N-shaped nullclines of ULK1 and AMPK intersect at an unstable point, generating a limit cycle oscillation of the pre-activator (AMPK) and the activator (ULK1) (Figure 5B, upper panel). We assume that this periodic change of AMPK and ULK1 is able to make the autophagy initiation itself periodic.

Although the double-negative feedback loop between AMPK and mTOR resulted in a limit cycle oscillation upon cellular stress (such as mimicking starvation), in the absence of mTOR (i.e., rapamycin treatment), it could not generate the periodic replication of autophagy (Figure 5B, lower panel), while the absence of the activator’s antagonist (mTOR) completely eliminates the double-negative feedback loops in the autophagy control network.

Since periodic autophagy induction has also been experimentally demonstrated during rapamycin treatment [33,34], the regulatory triangle of AMPK-ULK1-mTOR requires further extension.

### 2.5. The Additional Protein Has Direct or Indirect Connections with Its Targets and Regulators

Our theoretical analysis revealed that for proper oscillatory characteristics of autophagy induction, the AMPK-ULK1-mTOR control network requires an extra regulatory protein, called REG in our nomenclature (Figure 1).

**Figure 6:**
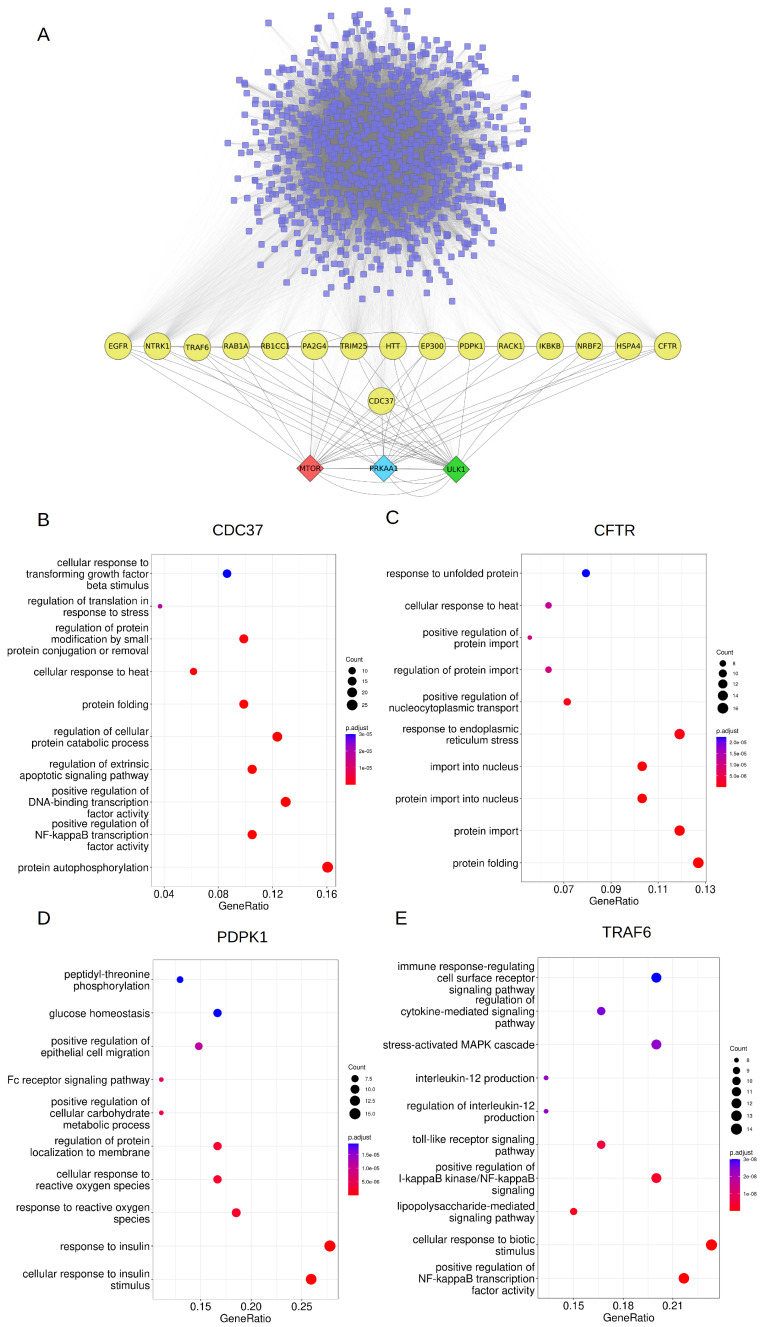
Results of network analysis. (**A**) Network image of filtered dataset from AutophagyNet. The AMPK-mTOR-ULK1 triangle is shown at the bottom, with proteins meeting all (CDC37) or two of the defined criteria. Above, the network of indirect regulators is shown. (**B**) Top 10 significantly enriched biological functions of the upstream network of CDC37. (**C**) Top 10 significantly enriched biological functions of the upstream network of CFTR. (**D**) Top 10 significantly enriched biological functions of the upstream network of PDPK1. (**E**) Top 10 significantly enriched biological functions of the upstream network of TRAF6.

From our analysis of AutophagyNet data, we have identified CDC37 to be the sole protein fulfilling all three points of the criteria: (1) getting induced by AMPK; (2) having a positive effect on ULK1; (3) having a negative effect on mTOR. Furthermore, we have identified 15 proteins to fulfil two of the three points of the criteria (Figure 6A). Functional analysis of the regulatory network of these proteins reveals that external-signal-related functions—such as response to reactive oxygen species or stress signals—are prominent in the regulatory network of the AMPK-ULK1-mTOR regulatory triangle. Analysis of the upstream regulatory network of CDC37 shows functions related to cell proliferation and reorganisation, which are crucial mechanisms required for autophagy (Figure 6B). Dysfunction of CFTR leads to cystic fibrosis, which is also characterised by defective autophagy, lipid metabolism and immune response [39]. Its regulators are involved in the response to endoplasmic reticulum stress and unfolded proteins, which indicate the importance of stress signals in autophagy induction (Figure 6C). PDPK1, previously shown to have a role in prostate cancer cell survival [40], also fulfils two of the set criteria. It is also known that PDPK1 plays a role in the regulation of autophagosome biogenesis [41]; therefore, understanding the function involved in its regulatory network can reveal important aspects of autophagy regulation as well. As in many of the 15 REG proteins, stress-related functions are also prominent in its regulatory network. Furthermore, immune-related functions such as the Fc receptor signalling pathway are also involved in autophagy regulation by the PDPK1 network (Figure 6D). Stress- and immune-related pathways can also be found in the TRAF6 network (Figure 6E), further proving the importance of these pathways in autophagy modulation. The abovementioned functions play a role in many diseases; therefore, experimental verification of this analysis can point out previously unknown connections between autophagy and pathological conditions.

### 2.6. The Additional Protein Acts like a Key Regulator of Autophagy Induction upon Cellular Stress

**Figure 7:**
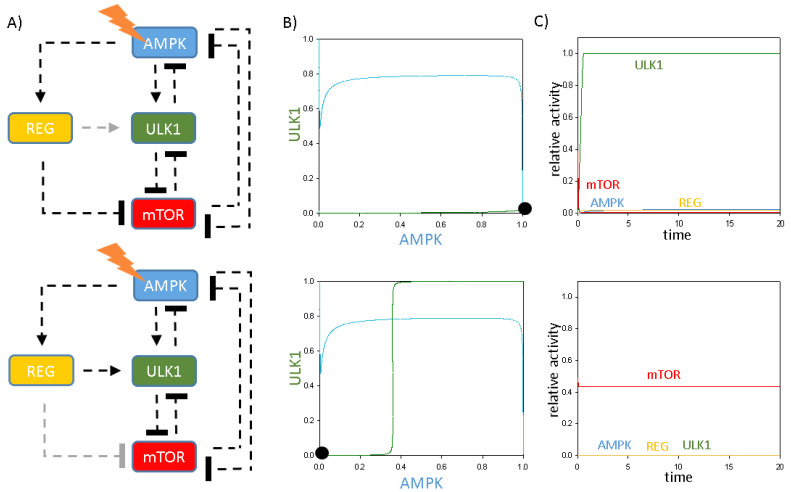
The extra protein acts like a key “regulator” of autophagy induction upon cellular stress. (**A**) The simple wiring diagram of autophagy induction controlled by an extra protein upon cellular stress when (**upper panel**) REG -> ULK1 (kaulk’ = 0) or (**lower panel**) REG -| mTOR (kimtor”’ = 0) regulatory connections are missing (see grey lines). Dashed lines show how the molecules can influence each other. Blocked end lines denote inhibition. (**B**) Phase plane diagrams are plotted upon cellular stress when (**upper panel**) REG -> ULK1 (kaulk’ = 0) or (**lower panel**) REG -| mTOR (kimtor”’ = 0) regulatory connections are missing. The balance curves of ULK1 (green) and mTOR (red) are plotted. Intersections of nullclines represent the stable (filled circle) and unstable (unfilled circle) steady states. Trajectories are depicted with dotted grey lines. (**C**) The temporal dynamics is simulated under cellular stress when (**upper panel**) REG -> ULK1 (kaulk’ = 0) or (**lower panel**) REG -| mTOR (kimtor”’ = 0) regulatory connections are missing. The relative activity of mTOR, AMPK, ULK1 and REG is shown.

We claim here that REG is crucial to control periodic repetition of self-cannibalism induction upon various stress events. To further analyse the importance of the “regulator”, we tested mutant phenotypes when the effects of REG were diminished systematically (Figure 7). If REG cannot enhance ULK1, the ULK1 nullcline remains flat, resulting in one stable intersection of AMPK and ULK1 nullclines under cellular stress, when AMPK is active and ULK1 is inactive (Figure 7A,B, upper panel). Simulated time course also shows that although AMKP is active and mTOR gets down-regulated, autophagy initiation cannot turn on in the absence of ULK1 activation (Figure 7C, upper panel).

The absence of REG-dependent mTOR inhibition also has a drastic effect on autophagy-dependent survival upon cellular stress; specifically, the nullclines of AMPK and ULK1 have one stable steady state upon cellular stress, where both of them are inactive (Figure 7A,B, lower panel). Time course simulation has confirmed that mTOR remains high in the absence of REG -| mTOR; therefore, both ULK1 and AMPK remain in their inactive states and no autophagy induction is observed (Figure 7C, lower panel). These results imply that AMPK alone is not sufficient to push mTOR into its inactive state, where it also needs the help of REG to down-regulate mTOR. Besides, in the absence of ULK1, the cell cannot turn on autophagy initiation upon cellular stress.

Together, these results suggest that both REG -> ULK1 and REG -| mTOR connections are essential for proper activation of autophagy upon cellular stress, confirming that REG is the key regulator of activation of autophagy.

## 3. Discussion

Autophagy is an essential mechanism for cell survival upon various intracellular or extracellular stress events (such as starvation or accumulation of damaged components), while the process is directly inhibited by the key regulator of cellular homeostasis, called mTOR. Autophagy allows the cell to get rid of all the harmful and/or unnecessary components and then synthesise what it really needs; however, uncontrolled autophagy might also have severe consequences. We have proved recently, both experimentally and theoretically, that the periodic repetition of on and off of autophagy is crucial for cellular survival upon cellular stress. Here, our systems biology analysis has shown that not only does a toggle switch based on the mTOR-ULK1 double-negative feedback loop control the induction of autophagy but also the AMPK-ULK1 negative feedback loop is needed in the regulatory system to explain the periodic induction of autophagy (Figure 1 and Figure 2). We envision that these double-negative and negative feedback loops together control the ability of the cell to induce sustained autophagy under high stress; however, under transient stress levels, the induction of autophagy becomes periodic, allowing the cell to use the macromolecules generated (Figure 2).

We have also shown here that a key control molecule (called “regulator”—REG) is required for autophagy induction to be properly regulated. This REG not only delays the negative feedback loop between AMPK and ULK1—thus, allowing limit cycle oscillation of autophagy induction—but also helps to switch the cell from a physiological state to autophagy in the presence of cellular stress by inhibiting mTOR (Figure 1, Figure 2 and Figure 7). Using AutophagyNet, we have identified 16 such “regulators”, which have been experimentally demonstrated to fulfil at least two of the following criteria: (1) getting induced by AMPK; (2) having a positive effect on ULK1; (3) having a negative effect on mTOR (see Figure 6 and Supplementary Figure S3). By reconstructing the upstream regulatory network of these regulators, we show that cellular stress- and immune-related functions play an important role in the modulation of autophagy. Our analysis depicted that Cdc37 has all three regulatory connections; in addition, it is directly activated by AMPK and also directly regulates ULK1 positively and mTOR negatively. It was recently shown that oncogene Cdc37 as a co-chaperone assists the molecular chaperone activity of Hsp90, controlling a large number of intracellular signalling networks [42]. It was also proved that high levels of Cdc37 are observed in some types of cancers [43,44]. According to our regulatory network, we claim that Cdc37 acts like a “regulator” of autophagy and its artificially high level results in down-regulation of mTOR and hyper-activation of autophagy. There is already clinical evidence that uncontrolled autophagy in melanoma and hepatocellular carcinoma leads to early metastasis and poor prognosis [45,46]. Our results further confirm that Cdc37 might be a good candidate for broad-spectrum molecular cancer therapy.

Cystic fibrosis transmembrane conductance regulator (CFTR) dysfunction leads to cystic fibrosis [47]. CFTR dysfunction also leads to disruption of autophagy [48]. In our analysis, we show that CFTR is directly activated by AMPK, while negatively regulating mTOR, therefore fulfilling two of the three criteria. Functional analysis of the upstream network of CFTR shows that CFTR-related autophagy modulation is crucial for cell reorganisation and response to cellular stress signals.

TNF receptor associated factor 6 (TRAF6) plays an important role in tumour invasion and metastasis [49], and has also been associated with regulating autophagy induction [50,51]. We have shown that TRAF6 directly regulates both ULK1 and mTOR. Functional analysis of regulators of TRAF6 (indirect regulators of autophagy) reveal the importance of innate immune- and stress-related pathways in autophagy modulation (Figure 6E). The role of interleukin 12 (IL-12) has been described in many infectious and inflammatory conditions [52,53]. IL-12 has also been described to induce autophagy through AMPK [54]. Interleukin-12-related functions in the indirect regulators of autophagy converging on TRAF6 indicate this protein as a potentially important target in disease therapies.

Oxidative-stress-related functions in our results further highlight the importance of autophagy induction in stress response. Hypoxia can lead to an increase in reactive oxygen species (ROS) generation in the cell, which are known to trigger a negative feedback loop through autophagy, to eliminate ROS [55,56]. Therefore, pathways of responses to reactive oxygen species in networks of multiple REG proteins (e.g., PDPK1, HSPA4, Figure 6, Supplementary Figure S1) could further indicate a connection between the regulation of autophagy induction and hypoxia. Furthermore, it is known that together with HIF-1, mTOR is also involved in the coordination of hypoxia-induced autophagy [57]. Taken together, our results further confirm the importance of the AMPK-ULK1-mTOR triangle in hypoxia response.

## 4. Mathematical Models and Methods

### 4.1. Mathematical Modelling

With ordinary differential equations (ODE), temporal changes in a biological regulatory network can be described. A generic differential equation of a regulatory component is composed of two parts: production and consumption terms. In a cellular protein–protein regulatory network, the production can be given by protein synthesis (i.e., transcription and translation) and/or an activation (i.e., post-translational modification) term, while the consumption can be given by protein degradation and/or an inactivation term. Usually, synthesis and degradation reactions are described by mass action kinetics, whereas protein activity can be described either by mass action or Michaelis–Menten kinetics. Solving a set of non-linear ODEs gives the time evolution of the relative protein concentration/activity (time courses).

In the model on which the manuscript is based, the relationships between ULK1, mTORC1 and AMPK are described and the dynamical behaviour of the control network are investigated upon various cellular stresses. They experimentally proved that autophagy could oscillate in case of mTORC1 down-regulation [12,33]. To generate a sustained oscillation in molecular systems, the following conditions have to be met: (1) negative feedback is present; (2) the negative feedback is sufficiently delayed; (3) the kinetic rate laws are sufficiently “nonlinear”; (4) the reactions occur in appropriate time scales [58]. The authors verified the important negative feedback loops of the ULK1-AMPK-mTORC1 regulatory triangle and also concluded that the model could only oscillate if a time delay was present in the system [12,33].

Here, our addition to the model is a delayed effect of AMPK on the system through a new regulatory element (“regulator”—REG). The extended model presented here consists of 4 variables—the relative activity/concentrations of ULK1, AMPK, mTORC1 and REG proteins—and 25 parameters. The parameter values and the detailed system of equations can be found in the supplementary documents (Supplementary Table S1). Besides dynamical systems theory techniques, global sensitivity analysis was also used for the analysis of the model. The methods used are detailed in the Supplementary Materials.

### 4.2. Network Analysis Methods

Direct and additional protein regulatory layers were downloaded from the AutophagyNet database https://autophagynet.org accessed on 20 February 2023. From this dataset, interactors were selected that met at least two of the defined criteria: (1) getting induced by AMPK; (2) having a positive effect on ULK1; (3) having a negative effect on mTORC1. Upstream regulators of these direct effectors were then filtered out from the dataset. The final effect of a regulatory stream was kept the same, meaning an upstream regulator of a ULK1 activator (protein X) cannot be an inhibitor of said protein, since the desired criteria would not be achieved otherwise. Interactions of the resulting list of regulators were re-compiled and then visualised using Cytoscape [59]. Where possible, experimentally verified direction and signage annotations from AutophagyNet were taken into account. However, these were not available for all connections. In these cases, predicted signage and direction information was used, likewise from AutophagyNet. It is important to note that without experimental verification our results should be considered with circumspection. Individual networks for direct regulators of the AMPK-mTORC1-ULK1 triangle were filtered so that only incoming, directed upstream regulators on a direct regulator were selected. For each network, over-representation analysis was run with ClusterProfiler (Supplementary Figure S3). Only significantly enriched functions were considered, defined by a q-value cutoff of 0.05. Results were visualised on dot plots in R.

## 5. Conclusions

The work presented here investigated the dynamical characteristic of autophagy induction upon various cellular stresses. A simple model was created with four components to analyse the importance of both the components and the feedback loops to manage an oscillatory characteristic of autophagy initiation upon various cellular stress events. A critical point of our model may be that it is oversimplified and the parameter values are only vague estimates based on previous studies; however, here, we successfully explore the minimum requirement for both the switch-like and periodic characteristics of autophagy induction. Furthermore, the sensitivity analysis also supports our hypothesis that some of AMPK’s effect on ULK1 is delayed through an intermediary regulatory component (Supplementary Figure S1). The used method for the Sensitivity analysis was the Sobol method. The method ranks the inputs to the model (in this case, the parameters) based on which input value causes the largest variance in the model output. The analysis suggests that the protein–protein interactions that most influence autophagy induction are AMPK inhibition by ULK1 and REG activation by AMPK.

To explore the oscillatory characteristic of the regulatory network upon cellular stress, the stress level was systematically increased; meanwhile, the presence of either active AMPK, mTOR and ULK1 was detected (Supplementary Figure S2). Each AMPK, mTOR and ULK1 bifurcation diagram contains a relatively wide range of oscillation with well-defined boundaries suggesting that the chosen parameter set closely approximates the biological system. We only investigate here the initiation steps of autophagy, and confirm its periodic induction via the mTOR-AMPK-ULK1 regulatory triangle, but we do not claim that oscillatory characteristics are observable for all the steps of autophagy (initiation, elongation, maturation, fusion and degradation). Our model is based on our best knowledge of the available literature, but it cannot be ignored that undiscovered links may change the picture. Other proteins may be identified in the future that meet the “regulator” criteria we have defined.

## Data Availability

Not applicable.

## Supplementary Materials

The following supporting information can be downloaded at: https://www.mdpi.com/article/10.3390/ijms24087671/s1, References [60,61] are cited in the supplementary materials.

## Abbreviations

The following abbreviations are used in this manuscript:

AMPK	5′ AMP-activated protein kinase
mTORC1	mammalian target of rapamycin complex 1
ULK1	Unc-51 like autophagy activating kinase 1
FKBP12	12-kDa FK506-binding protein
ODE	ordinary differential equation
CFTR	cystic fibrosis transmembrane conductance regulator
TRAF	TNF receptor associated factor
