# Supplementary material for: Oscillation of Autophagy Induction under Cellular Stress and What Lies behind It, a Systems Biology Study
Source: Int J Mol Sci. 2023 Apr 21;24(8):7671. doi: 10.3390/ijms24087671 (PMC10143760; doi:10.3390/ijms24087671)
Supplement: Supplementary file 1 [file ijms-24-07671-s001.zip › ijms-2346432-SI.pdf]

Supplementary Information
Oscillation of Autophagy Induction under Cellular Stress and What Lies behind It, a Systems Biology Study

1 Equations and parameters of ULK1 induced autophagy model

The variables of the dynamical system are the active form of the regulatory elements. The maximum concentrations of ULK1, mTOR, AMPK and REG are denoted as $ULK1_T$, $mTOR_T$, $AMPK_T$, REG_T respectively. The interaction between the variables is described by first order reactions, and Michaelis-Menten kinetics Equations S1-S4.

$$\frac{dULK1}{dt} = \frac{(k_{aulk} + k_{aulk1} * REG + k_{aulk2} * AMPK) * (ULK1_T - ULK1)}{J_{ulk} + ULK1_T - ULK1} - \frac{(k_{iulk} + k_{iulk1} * mTOR) * ULK1}{J_{ulk} + ULK1} \tag{S1}$$

$$\frac{dmTOR}{dt} = k_{amtor} * (mTORT - mTOR) - (k_{imtor} + k_{imtor1} * AMPK + k_{imtor2} * ULK1 + k_{imtor3} * REG) * mTOR \tag{S2}$$

$$\frac{dAMPK}{dt} = \frac{(k_{aak} + STARV) * (AMPK_T - AMPK}{(J_{ampk} + AMPK_T - AMP} - \frac{-(k_{iak} + k_{iak1} * ULK1 + k_{iak2} * mTOR) * AMPK}{J_{ampk} + AMPK} \tag{S3}$$

$$\frac{dREG}{dt} = \frac{(k_{areg} + k_{areg1*AMPK}) * (REG_T - REG)}{J_{reg} + REG_T - REG} - \frac{k_{ireg*REG}}{J_{reg} + REG} \tag{S4}$$

The parameter values and their effects is described in Table S1.

Table S1: Parameter values and their effects.

Parameter	Value	Dimension	Effect
$ULK1_T$	1	[-]	Maximum ammount of ULK1 in the system
$AMPK_T$	1	[-]	Maximum ammount of AMPK in the system
$mTOR_T$	1	[-]	Maximum ammount of mTOR in the system
REG_T	1	[-]	Maximum ammount of REG in the system
S	$0 < S < 1$	[-]	stress levels
k_{aulk}	0.101	min^{-1}	ULK1 background activity
k_{aulk1}	25	min^{-1}	ULK1 activation by REG
k_{aulk2}	0.03	min^{-1}	ULK1 activation by AMPK
k_{iulk}	0.1	min^{-1}	Background inhibition of ULK1
k_{iulk1}	20	min^{-1}	Ulk1 inhibition by mTOR
J_{ULK}	0.001	[-]	ULK1's Michaleis-Menten constant
k_{aak}	0.2	min^{-1}	AMPK background activation
k_{iak}	0.1	min^{-1}	Background inhibition of AMPK
k_{iak1}	3	min^{-1}	AMPK inactivation by ULK1
k_{iak2}	10	min^{-1}	AMPK inactivation by mTOR
J_{AMPK}	0.01	[-]	AMPK's Michaelis-Menten constant
k_{amtor}	0.0175	min^{-1}	mTOR background activation
k_{imtor}	0.0125	min^{-1}	Background inhibition of mTOR
k_{imtor1}	10	min^{-1}	mTOR inhibition by AMPK
k_{imtor2}	0.1	min^{-1}	mTOR inhibition by ULK1
k_{imtor3}	0.1	min^{-1}	mTOR inhibition by mTOR
J_{REG}	0.02	[-]	REG's Michaelis-Menten constant
k_{areg}	0.01	min^{-1}	Background activation of REG
k_{areg1}	10	min^{-1}	REG activation by AMPK
k_{ireg}	20	min^{-1}	Background inhibition of REG

2 Global sensitivity analysis

Since our parameter values are only tuned by comparsion of the model output to data, the parameters have high uncertainty. Global sensitivity analysis makes it possible to study the effects of this uncertainty on the model output Sumner (2010).

In this study we used Julia's eFAST method to calculate the first and total Sobol indices of the model parameters Dixit and Rackauckas (2022), which is a variance based method. Let

$$Y = f(X_1, X_2, ..., X_n) \tag{S5}$$

then the first order Sobol index of equation S5's ith input is defined as

$$S_i = \frac{Var_{X_i}(E_{X\sim i}(Y|X_i))}{Var(Y)} \tag{S6}$$

similarly the total Sobol index if the ith model input is defined as

$$S_{T_i} = 1 - \frac{Var_{X\sim i}(E_{X_i}(Y|X_{\sim i}))}{Var(Y)} \tag{S7}$$

That is, the greater the value of the parameter's Sobol indices (the greater the variance it causes), the greater its effect on the system.

In the analysis the parameter values were varied uniformly around their assumed value by ±50%. In this range the system remained numerically stable, and the main qualitative dynamic features are kept as well.

Figure S1 shows the impact of the parameters on the ULK1 level in ascending order. The analysis suggests that ULK1 level is most dependent on the stress levels in the system and the strength of the $ULK1 - |AMPK$ interaction. The fourth highest Sobol index belongs to the $AMPK- > REG$ reaction, which suggests that the impact of this reaction on the system is significant.

3 Mathematical codes for computational simulations

The mathematical models presented on the Figures were computed numerically using XPP-AUT, which is freely available from it's website. All the used codes for the numerical analysis are available on GitHub. The proteins levels/activities are given in arbitrary units (a.u).

4 Figures

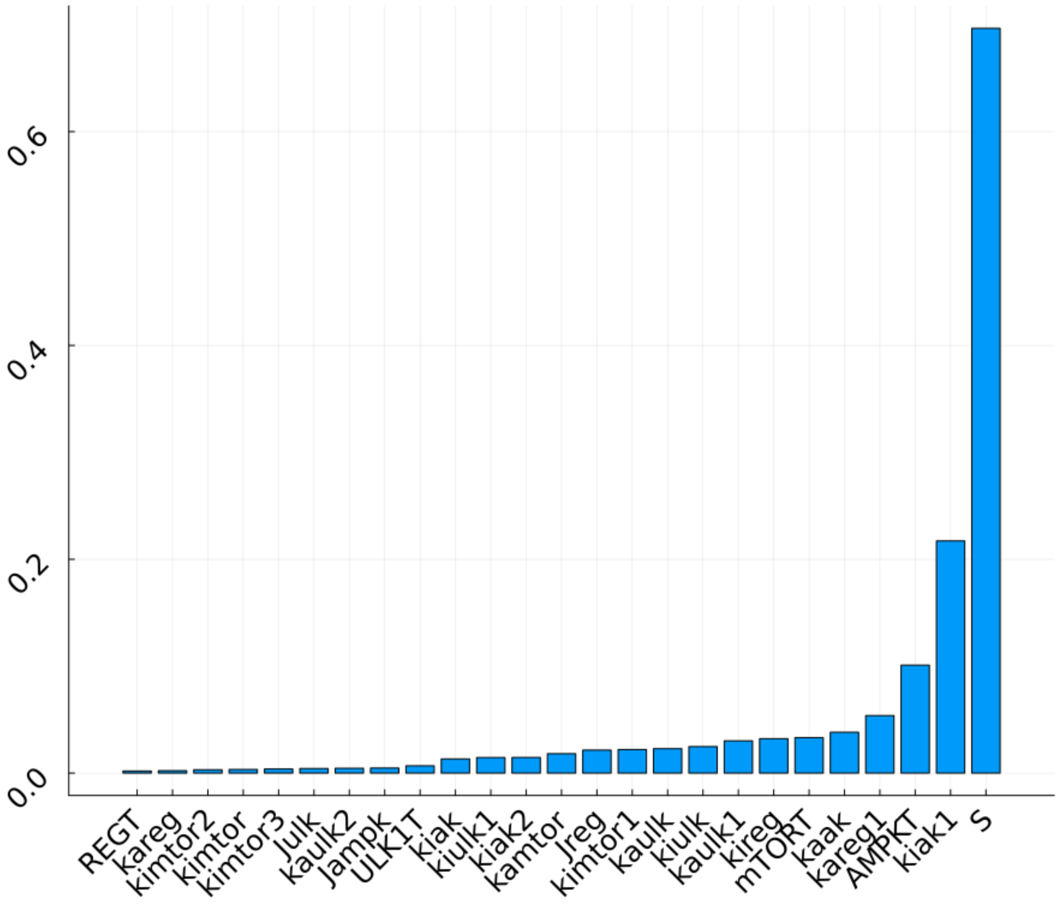

Figure S1: Total Sobol indices of ULK1 model output. The results suggest that the stress level has the most impact on the ULK1 levels (parameter denoted with **S**. Then the AMPK inhibition by ULK1 (**kiak1**), total possible AMPK levels (**AMPKT**) and the activation of REG by AMPK (**kareg1**).

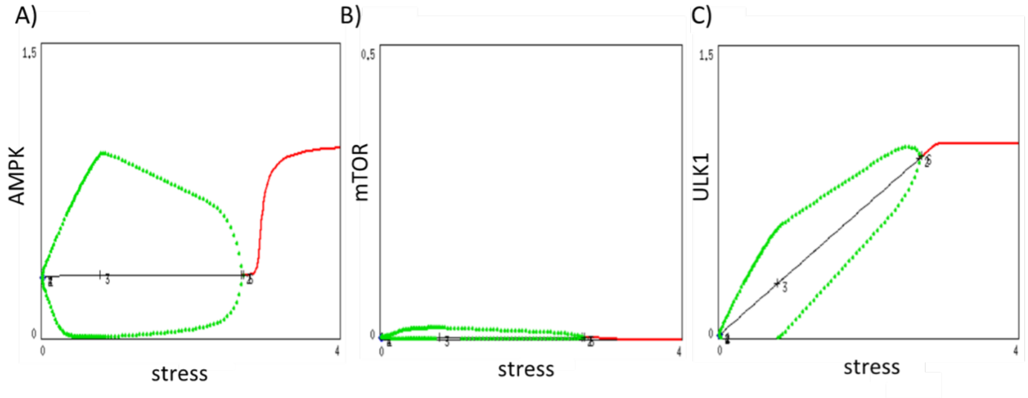

Figure S2: The oscillatory characteristic of the oxidative stress response mechanism. The bifurcation diagrams of **A)** AMPK, **B)** mTOR and **C)** ULK1. The signal response curves are shown with respect to the increasing stress level. Solid red lines denote stable states, while solid black line denotes the unstable state. Green dots around the unstable states represent oscillatory behaviour.

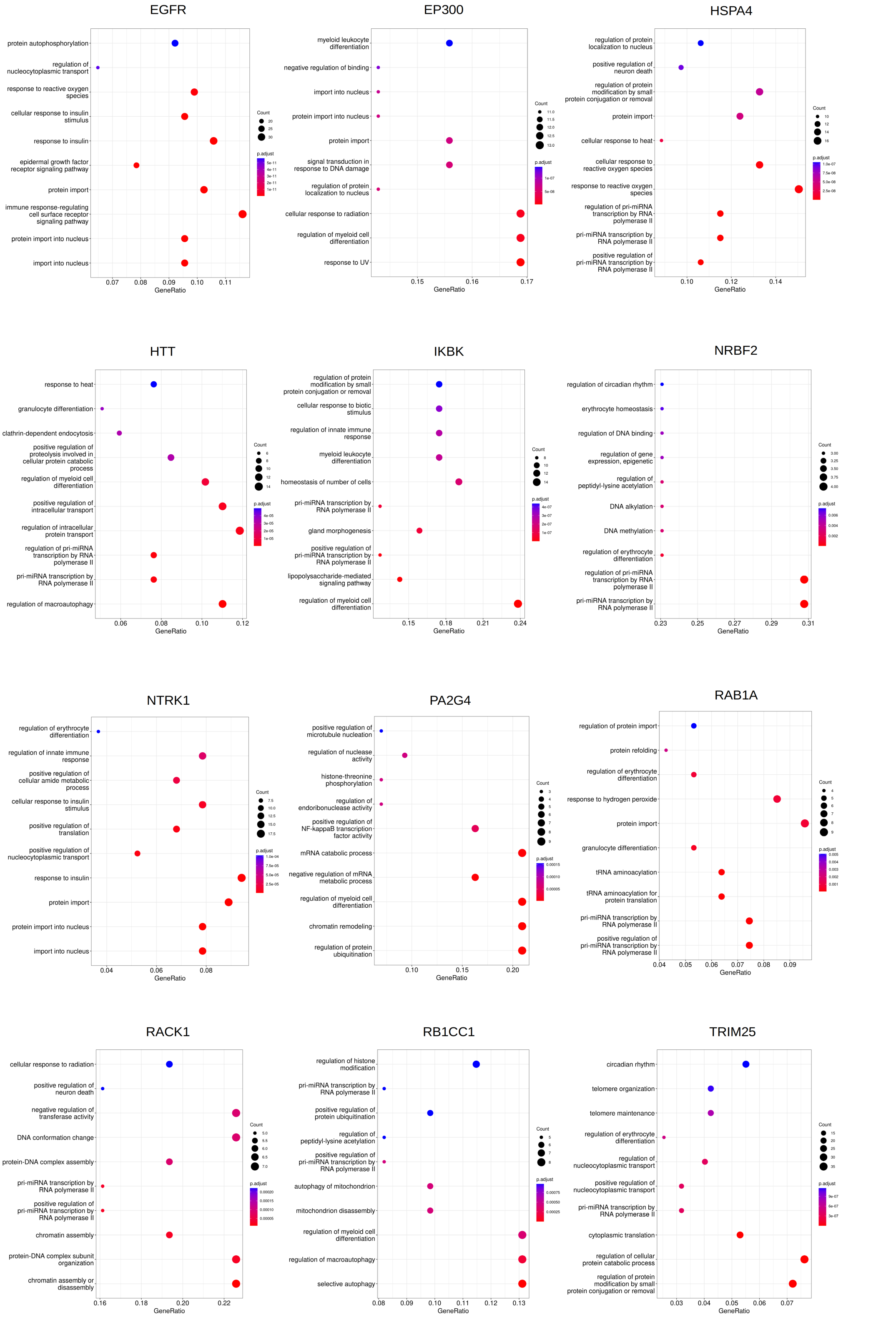

Figure S3: Top 10 significantly enriched biological functions of upstream networks of additional proteins completing two criteria.

References

Dixit, V. K. and C. Rackauckas (2022). Globalsensitivity. jl: Performant and parallel global sensitivity analysis with julia. *Journal of Open Source Software 7*(76), 4561.

Sumner, T. (2010). *Sensitivity analysis in systems biology modelling and its application to a multi-scale model of blood glucose homeostasis.* Ph. D. thesis, UCL (University College London).
